# Construction and Verification of a Combined Hypoxia and Immune Index for Clear Cell Renal Cell Carcinoma

**DOI:** 10.3389/fgene.2022.711142

**Published:** 2022-02-09

**Authors:** Bin Wang, Lixiao Liu, Jinting Wu, Xiaolu Mao, Zhen Fang, Yingyu Chen, Wenfeng Li

**Affiliations:** ^1^ Department of Medical Oncology, the First Affiliated Hospital of Wenzhou Medical University, Wenzhou, China; ^2^ Department of Obstetrics and Gynecology, the First Affiliated Hospital of Wenzhou Medical University, Wenzhou, China; ^3^ Department of Neurosurgery, the First Affiliated Hospital of Wenzhou Medical University, Wenzhou, China

**Keywords:** clear cell renal cell carcinoma, hypoxia, immune, TCGA, prognosis, signature, qRT-PCR

## Abstract

Clear cell renal cell carcinoma (ccRCC) is one of the most aggressive malignancies in humans. Hypoxia-related genes are now recognized as a reflection of poor prognosis in cancer patients with cancer. Meanwhile, immune-related genes play an important role in the occurrence and progression of ccRCC. Nevertheless, reliable prognostic indicators based on hypoxia and immune status have not been well established in ccRCC. The aims of this study were to develop a new gene signature model using bioinformatics and open databases and to validate its prognostic value in ccRCC. The data used for the model structure can be accessed from The Cancer Genome Atlas database. Univariate, least absolute shrinkage and selection operator (LASSO), and multivariate Cox regression analyses were used to identify the hypoxia- and immune-related genes associated with prognostic risk, which were used to develop a characteristic model of prognostic risk. Kaplan-Meier and receiver-operating characteristic curve analyses were performed as well as independent prognostic factor analyses and correlation analyses of clinical characteristics in both the training and validation cohorts. In addition, differences in tumor immune cell infiltrates were compared between the high and low risk groups. Overall, 30 hypoxia- and immune-related genes were identified, and five hypoxia- and immune-related genes (*EPO*, *PLAUR*, *TEK*, *TGFA*, *TGFB1*) were ultimately selected. Survival analysis showed that the high-risk score on the hypoxia- and immune-related gene signature was significantly associated with adverse survival outcomes. Furthermore, clinical ccRCC samples from our medical center were used to validate the differential expression of the five genes in tumor tissue compared to normal tissue through quantitative real-time polymerase chain reaction (qRT-PCR). However, more clinical trials are needed to confirm these results, and future experimental studies must verify the potential mechanism behind the predictive value of the hypoxia- and immune-related gene signature.

## Introduction

Renal cell carcinoma (RCC) is one of the most common malignant tumors of the urinary system, approximately 4% (73,750 new cases) of newly diagnosed carcinomas in United States (Siegel et al., 2020). In 2020, the global incidence of RCC was 431,000 patient cases, and the death toll was 179,000 people, which represented 1.8% of the global death toll from cancer; morbidity and mortality rates are still increasing (Padala et al., 2020). Clear cell RCC (ccRCC) accounts for approximately 75–80% of the pathological types of RCC (Ricketts et al., 2018; Vuong et al., 2019). Because it is insensitive to radiotherapy and chemotherapy, treatment of metastatic RCC remains poorly effective (Pal and Agarwal, 2016; Lieder et al., 2017; Lara and Evans, 2019). The primary treatment for early ccRCC is surgery, whereas chemotherapy, targeted therapy (tyrosine kinase inhibitors and mTOR inhibitors), and immunotherapy are the preferred treatments for advanced ccRCC (Vermassen et al., 2017; Atkins and Tannir, 2018; Chen et al., 2019). However, drug resistance after targeted therapy and limitations of immunotherapy impair patients’ long-term outcomes (Duensing and Hohenfellner, 2016; Xu et al., 2020).

Hypoxia-related mechanisms have long been considered markers of cancer signaling pathways (Jing et al., 2019). The hypoxic tumor microenvironment is closely associated with poor prognosis and poor survival (Gilkes et al., 2014). The fast propagation of tumor cells and the lack of blood supply lead to low oxygen levels within the tumor, which can lead to an anoxic focus. The genes with expression changes triggered under this condition are called hypoxia-related genes (HRGs). In solid tumors, tumor cells express hypoxia-inducible factor 1 (HIF-1), which persuades the expression of factors involved in tumorigenesis, including extracellular matrix remodeling, angiogenesis, cell migration, drug resistance, and tumor stem cell maintenance (Hajizadeh et al., 2019). A few studies have shown that hypoxia in tumor cells can promote angiogenesis, glycolysis, cell invasion, cell survival, and immune escape and eventually can lead to tumorigenesis and metastasis (Lee et al., 2019; Luo and Wang, 2019). The predictive power of HRGs in the prognosis of major malignancies (lung cancer or gastric cancer) has been well demonstrated (de Heer et al., 2020; Wang et al., 2021).

Currently, it is believed that the loss of immune cell function in the tumor microenvironment is one of the important mechanisms for malignant tumors to escape from the human immune system (Lawson et al., 2020). Current studies have shown that immune-related genes (IRGs) play an vital role in the development of RCC (Xu et al., 2019; Lawson et al., 2020). There is evidence that high levels of activated CD8^+^ T cells are associated with better prognosis in many cancers, including kidney cancer (Youngblood et al., 2017; Yao et al., 2018). In a retrospective analysis of the S-Trac trial using adjuvant sunitinib in high-risk patients with renal cancer, the number of CD8^+^ T-cell infiltrates in tumor samples highly correlated with survival prognosis in the sunitinib group (George et al., 2018). Interestingly, direct and indirect interactions between hypoxia and immune status have been found in the RCC microenvironment (Samanta and Semenza, 2018). In RCC, the EGLN/HIF signaling axis promotes tumorigenesis by altering the function of various components of the tumor microenvironment, including cancer-associated fibroblasts, endothelial cells, and immune cells (Huang et al., 2017).

So far, the relationship between the expression of hypoxia- and immune-related genes and ccRCC has not been studied in detail. In this study, a risk scoring model based on five hypoxia- and immune-related genes was constructed and validated using a public database to individualize prognosis in patients with ccRCC. In addition, the model was combined with clinical features to improve the accuracy of overall survival prediction. Differences in tumor immune cell infiltration between the high and low risk groups were also analyzed.

## Materials and Methods

### Data Acquisition and Analysis

**FIGURE 1:**
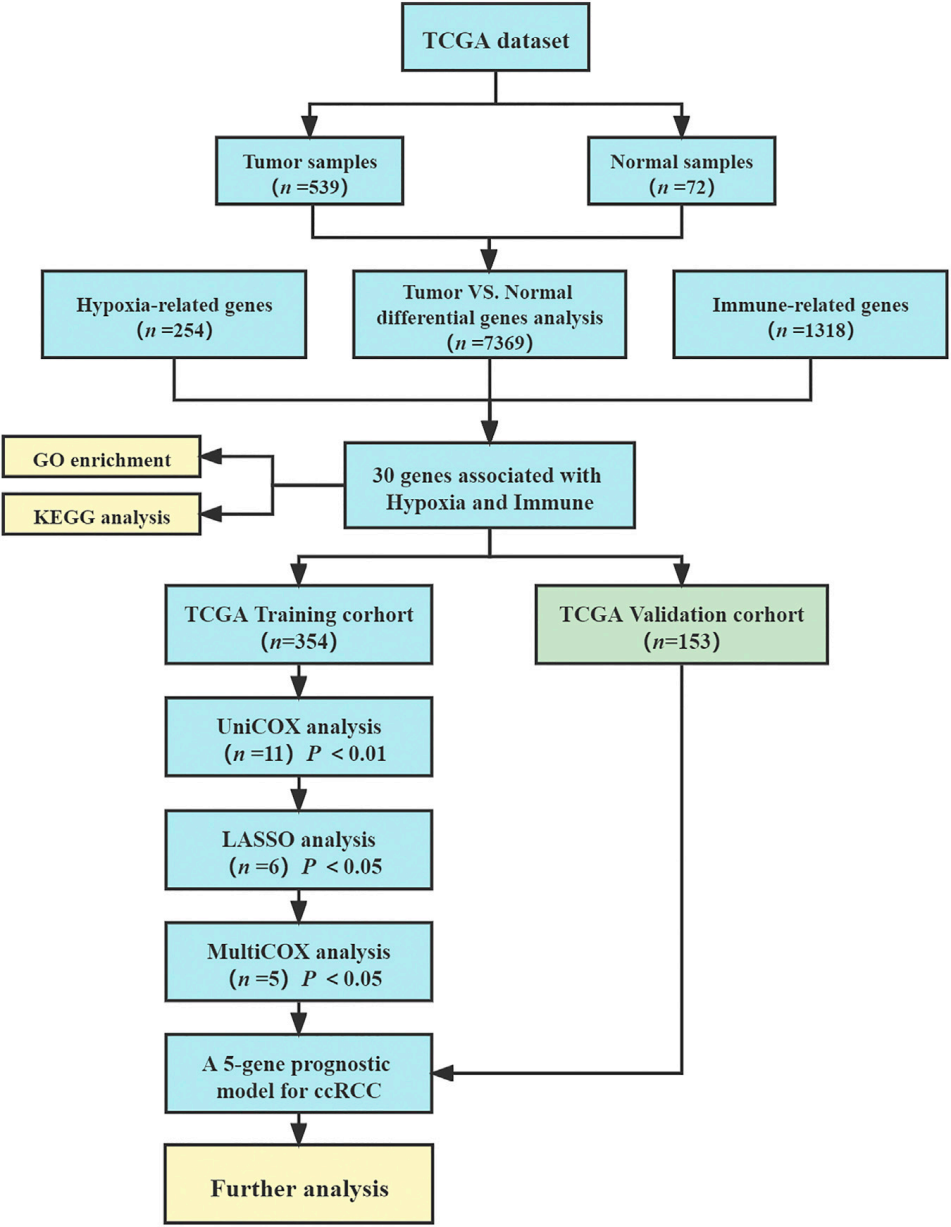
Flow chart.

The flow chart of this study is shown in Figure 1. We collected the gene expression data in the database of The Cancer Genome Atlas Program (TCGA-KIRC, https://portal.gdc.cancer.gov) (Liu et al., 2018). The research included the data of all 539 ccRCC tumor samples and 72 normal kidney samples. Clinical information of ccRCC patients was downloaded from TCGA-KIRC dataset, including age, gender, survival status, follow-up time, tumor grade, tumor stage, TNM stage. Then, patients with follow-up time less than 30 days and incomplete information were excluded, and 507 ccRCC patients were included in the model construction and survival analysis. 254 hypoxia-related genes were collected from HARRIS_HYPOXIA.gmt and WINTER_HYPOXIA_METAGENE.gmt by gene aggregation analysis (GSEA, http://www.gsea-msigdb.org/gsea) (Harris 2002; Subramanian et al., 2005; Winter et al., 2007). In addition, 1,318 IRGs were derived from IMMPORT database (Tian et al., 2020) (https://www.immport.org/home).

### Dentifying Differentially Expressed HRGs and IRGs in Clear Cell Renal Cell Carcinoma

To identify the differentially expressed genes (DEGs) between tumor and normal samples, the “limma” package in R was used to process the mRNA sequencing data (Ritchie et al., 2015) and visualized by “pheatmap” and “vioplot”packages in R (Cheng et al., 2021). The original data were preprocessed and standardized, and then 539 tumor samples were compared with 72 normal samples. The screening criteria for differential genes was false discovery rate (FDR) < 0.05, *p* value < 0.05 and |logFC| > 1. Venn diagrams (Jia et al., 2021) are used for graphical depiction of the unions, intersections and distinctions among DEGs, HRGs and IRGs.

### Pathway Enrichment Analysis to Identify Molecular Functions

To better understand the function of all hypoxia- and immune-related genes, we performed pathway enrichment analysis on these genes. We used the “clusterProfiler” package in R (Yu et al., 2012) to analyze the signaling path–related genes through the Kyoto Encyclopedia of Genes and Genomes (KEGG) (Kanehisa and Goto, 2000) database and to analyze their biologic processes, molecular functions, and cellular components through the R Gene Ontology (GO) database program (Gene Ontology Consortium, 2015). The aim was to determine whether the genes screened were indeed involved in hypoxia and immunity.

### Hypoxia- and Immune-Related Gene Prognosis Model Construction

We used a univariate Cox model to analyze the relationship between the expression levels of HRGs and IRGs and the overall survival of patients with ccRCC. Univariate Cox regression analyses were used to calculate the hazard ratio (HR) and 95% confidence intervals (CI) to identify genes associated with over-all survival (van Dijk et al., 2008). Using *p* value < 0.01 as the cutoff for identifying relevant genes, we selected survival-related genes. To avoid gene abundance fitting, we used the “glmnet” package to perform LASSO regression to screen for genes with higher correlation (Friedman et al., 2010). Finally, we used multivariate Cox regression analysis to determine the optimal prognostic indicators of the model (Li et al., 2020). The prognostic risk score model was established as follows:

$$Risk\;score\;=\overset{n}{\underset{i=1}{\sum }}\beta_{i}G_{i}$$

( $\beta_{i}$ is the coefficient of the gene i in multivariate Cox analysis; $G_{i}\;$ represents the expression value of gene i ; n is the number of genes in the signature) (Cai et al., 2020).

### Survival Analysis and ROC Curve

We used univariate Cox regression analysis to verify the influence of individual clinicopathologic factors on prognosis (van Dijk et al., 2008). In this study, all samples were reclassified into appropriate subgroups based on age, sex, and stage. We then collected mean risk scores for different subgroups and performed survival analyses to verify the validity of the predictive prognostic model. We used Kaplan-Meier survival analysis to compare prognostic power between subgroups using the “survival” and the “survminer” packages in R (Heagerty et al., 2000). The Receiver Operating Characteristic Curve (ROC) curves were compared to investigate the accuracy, sensitivity, and specificity of the model (Heagerty et al., 2000).

### Validation Cohort Analysis

The ccRCC samples obtained from TCGA were randomly divided into two groups, the training cohort (*n* = 354) and the validation cohort (*n* = 153) (Supplementary Table S1). We used the same method described in section 2.5 for validation in the validation group.

### Immune Microenvironment Analysis

We downloaded immune cell infiltration tables for TCGA-listed tumors from TIMER (http://timer.comp-genomics.org) (Li et al., 2017)and CIBERSORT (https://cibersort.stanford.edu) (Newman et al., 2015). We analyzed the correlation between risk score and immune cells using “limma” and “ggpubr” packages (Cheng et al., 2021). Immunization differences between high and low risk groups were compared.

### Nomogram Construction and Validation

To develop a more convenient and qualitative predictive tool for ccRCC patients, we used the “rms” package and “survival” package in R (Liu et al., 2021) to establish a nomogram based on the factors that were determined to have independent predictive ability by the entire TCGA cohort after multivariate Cox analysis, and calibration curves were plotted for 2, 4, and 6 years to judge the precision of the nomogram (Iasonos et al., 2008).

### The Expression of Genes Was Verified by qRT-PCR

Six ccRCC tissues and normal kidney tissues were collected from the First Affiliated Hospital of Wenzhou Medical University. Total RNA was extracted from ccRCC samples and normal renal tissue samples using TRIzol reagent (Thermo Fisher Scientific, Waltham, MA, United States). Single-stranded cDNA was synthesized from 1 µg of total RNA using the PrimeScript RT Reagent Kit with gDNA Eraser (Takara Biotechnology Co. Ltd., Dalian, China). Reverse transcription quantitative PCR was applied to explore the mRNA expression of the hub genes using a 7500 PCR system (Thermo Fisher Scientific) (Zhou et al., 2021). The following cycling conditions were adopted: 95°C for 2 min, followed by 40 cycles of 95°C for 10 s and 60°C for 30 s. The qPCR assays were performed for each sample in a reaction volume of 10 μL. The 2^−ΔΔCt^ method was used to determine relative gene expression levels, and β-Actin was used as an internal control to normalize the data (Sun et al., 2021). The primers used in this study were provided by Sangon Biotech (Shanghai) Company and are shown in Supplementary Table S2. Data were analyzed using GraphPad Prism 8.0 Software (GraphPad Software Inc., La Jolla, CA, United States) (Huang et al., 2020), and *t*-test was used to test the differences between tumor and normal samples (*p* value < 0.05) (Katzendorn et al., 2021).

### Statistical Analyses

All analyses were performed using R version 4.0.5. Unless otherwise noted, *p* value < 0.05 was significant.

## Results

### Identification of Differentially Expressed Hypoxia-Related Genes and Immune-Related Genes in Clear Cell Renal Cell Carcinoma

**FIGURE 2:**
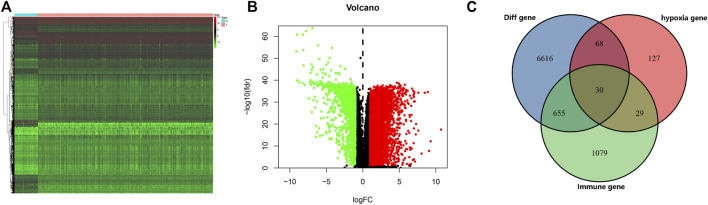
Screening of different expressed genes. **(A)** Heatmap of significantly different expressed genes. **(B)** Volcano map; green represents downregulated genes, and red represents upregulated of genes. **(C)** Venn plot of different expressed genes, hypoxia-related genes (HRGs), and immune-related genes (IRGs).

The database from TCGA included 539 tumor samples and 72 normal kidney samples. By comparing tumour and normal tissue samples, we finally screened 7,369 DEGs (Figure 2A, FDR value < 0.05, *p* value < 0.05 and |logFC| > 1). Compared with normal samples, 5,467 genes were upregulated and 1,903 genes were downregulated in tumor samples (Figure 2B). We collected data from HARRIS_HYPOXIA.gmt and WINTER_HYPOXIA_METAGENE.gmt to obtain a total of 254 HRGs. A total of 1,318 IRGs were derived from the IMMPORT database. Then, the intersection part of Venn diagram showed 30 common genes from the 7,369 DEGs, 254 HRGs and 1,318 IRGs (Figure 2C, Supplementary Table S3), which called the differentially expressed hypoxia- and immune-related genes were used for subsequent analysis.

### Functional Analysis of Hypoxia-Related Genes and Immune-Related Genes Pathways in Clear Cell Renal Cell Carcinoma

**FIGURE 3:**
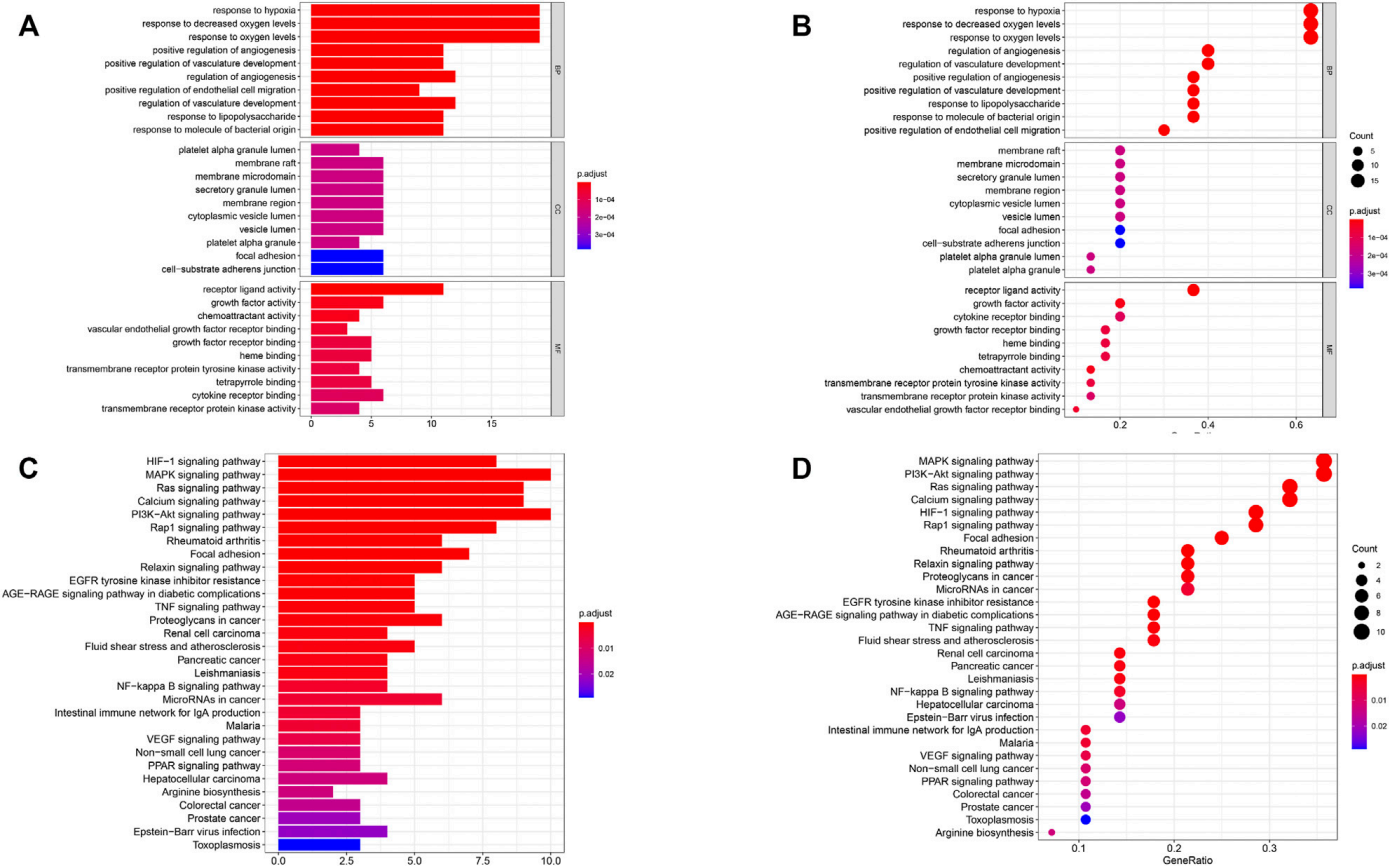
Functional pathway analysis. **(A, B)** Gene oncology (GO) pathway analyses. **(C, D)** Kyoto Encyclopedia of Genes and Genomes (KEGG) pathway analyses. BP: biologic process; CC: cellular component; MF: molecular function.

GO function analysis of these 30 genes showed that they were involved in hypoxia, bacterial origin molecules, lipopolysaccharides, regulation of vascular development, endothelial cell migration, angiogenesis, and vascular development (Figures 3A,B). KEGG pathway analysis showed that they were involved in rheumatoid arthritis–related pathways, the RAP1 signaling pathway, the PI3K/Akt signaling pathway, the calcium signaling pathway, the RAS signaling pathway, the MAPK signaling pathway, and the HIF-1 signaling pathway (Figures 3C,D). Based on these results, it has been shown that the genes we selected are indeed related to hypoxia and immunity.

### Construction and Verification of the Survival Model

**FIGURE 4:**
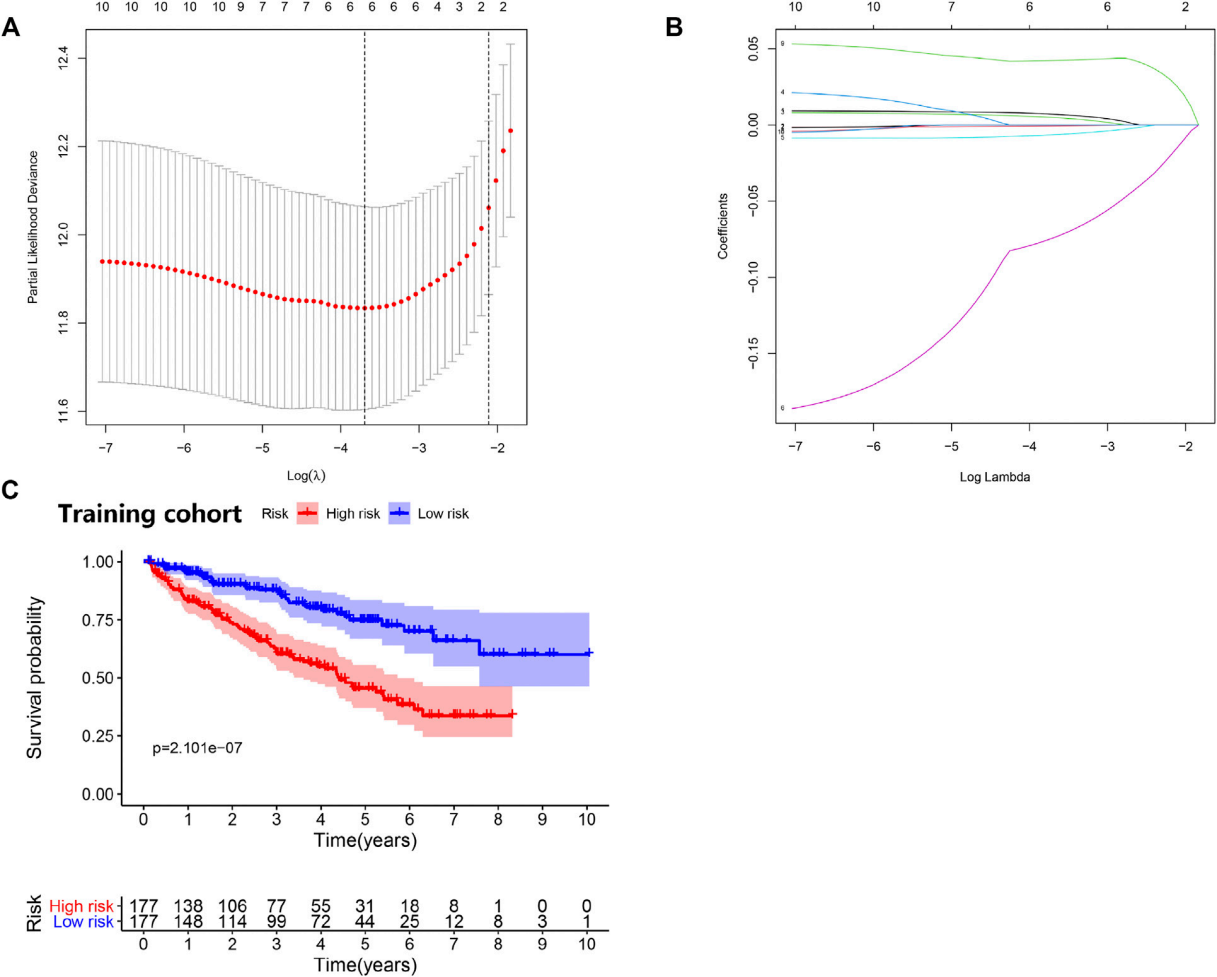
Prognostic risk model was constructed from the prognostic prediction in clear cell renal cell carcinoma (ccRCC). **(A)** Plots of the cross-validation error rates. **(B)** LASSO coefficient profiles of the prognostic risk model. **(C)** Survival curve for low- and high-risk subgroups.

**FIGURE 5:**
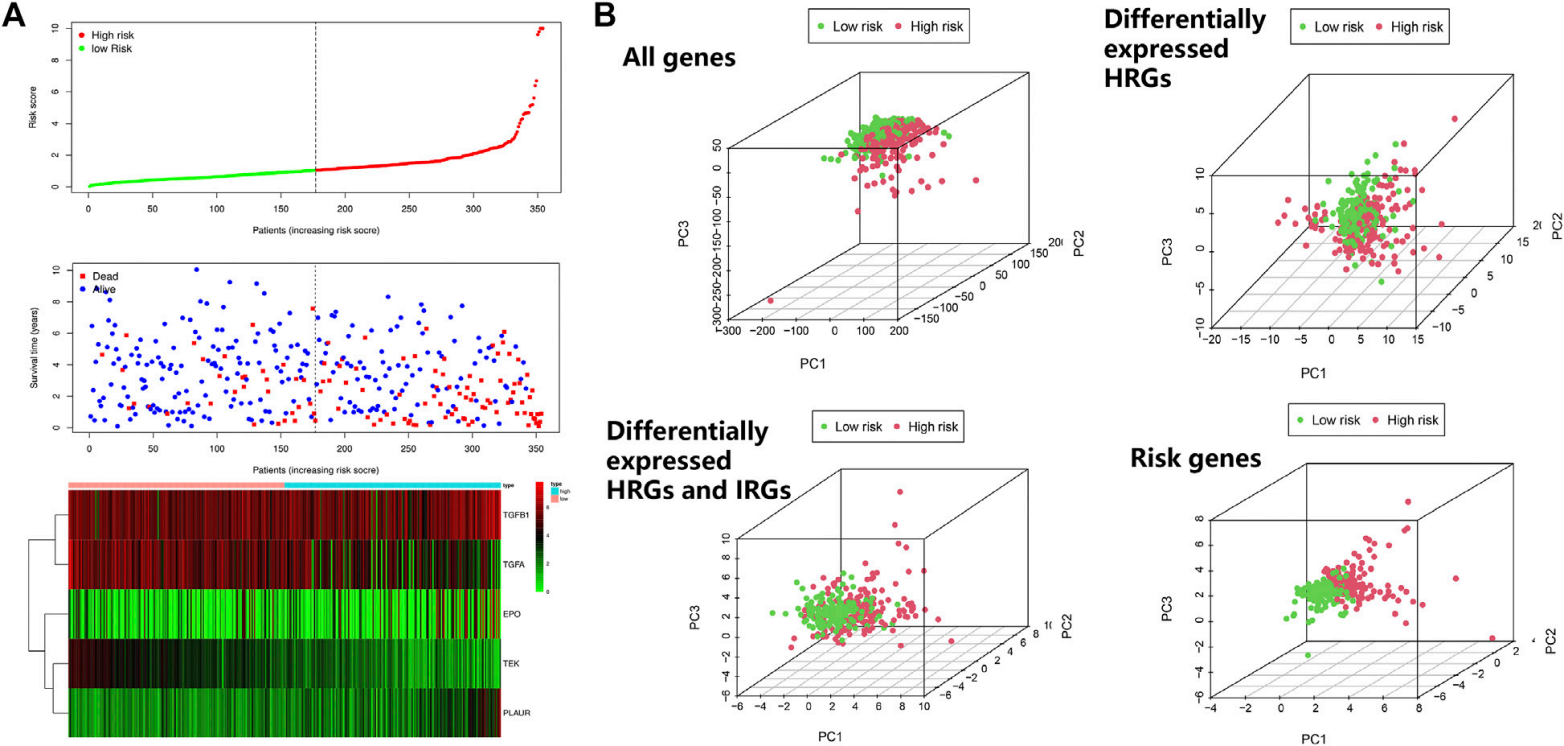
Risk score analysis of disease-specific survival–related prognostic models. **(A)** Risk score distribution, survival status, and expression heat map. **(B)** Principal components analysis (PCA)maps show the distribution of patients according to all genes (*n* = 56,753), differentially expressed HRGs (*n* = 98), differentially expressed HRGs and IRGs (*n* = 30), and risk genes (*n* = 5).

To search for new genetic biomarkers associated with prognosis in patients with ccRCC, we first performed univariate Cox analysis, in which 11 genes were significantly associated with overall survival (*p* value < 0.01, Supplementary Table S4). In addition, to screen for genes with higher correlation and to prevent overfitting of the model, we used LASSO regression analysis to reduce the number of candidate genes to six (*p* value < 0.05, Figures 4A,B, Supplementary Table S5). Five genes (*EPO*, *TGFB1*, *TGFA*, *TEK*, and *PLAUR*) independently related to overall survival were obtained by multivariate Cox analysis (*p* value < 0.05). Subsequently, we divided patients with ccRCC into low and high risk groups and examined the prognostic predictive performance of the new survival model consisting of five genetic risk characteristics. The hypoxia- and immune-related risk signature was constructed as follow: Risk score = 0.006828553 × Expression of EPO + 0.006828553 × Expression of TGFB1—0.011708366 Expression of TGFA—0.094278339 × Expression of TEK+ 0.044483942 × Expression of PLAUR. We evaluated the distribution of risk scores, survival information in the training cohort. As showcased in Figure 4C, with the gradual increase of the risk scores, the survival time of patients in high- and low-risk group gradually decreased, while the mortality rate gradually increased. As the risk score gradually increased, the expression levels of HRGs and IRGs in the samples gradually increased, and the overall survival rate showed a significant downward trend (Figure 5A). Principal components analysis (PCA) was also performed for all genes and for HRGs, HRGs and IRGs, and risk genes (Figure 5B). PCA showed that patients from different groups could be clearly grouped on the basis of signatures selected in all data sets. Analysis of the five hypoxia- and immune-related genes also showed high expression of *EPO*, *TGFB1*, *TGFA*, and *PLAUR*, but low expression of *TEK* in tumor samples (Table 1).

**TABLE 1:** Five hypoxia- and immune-related genes expressions in low and high risk groups.

Name	Coefficient	Type	Regulation	HR	95%CI	*p* value
EPO	0.00952	Protective	Up	1.010	1.002–1.017	0.0164402
TGFB1	0.00682	Protective	Up	1.007	0.999–1.015	0.0787113
TGFA	−0.01171	Risky	Up	0.988	0.979–0.998	0.0208366
TEK	−0.09428	Risky	Down	0.910	0.871–0.950	0.0000199
PLAUR	0.04448	Protective	Up	1.045	1.023–1.069	0.0000706

### Testing in the Validation Cohort

**FIGURE 6:**
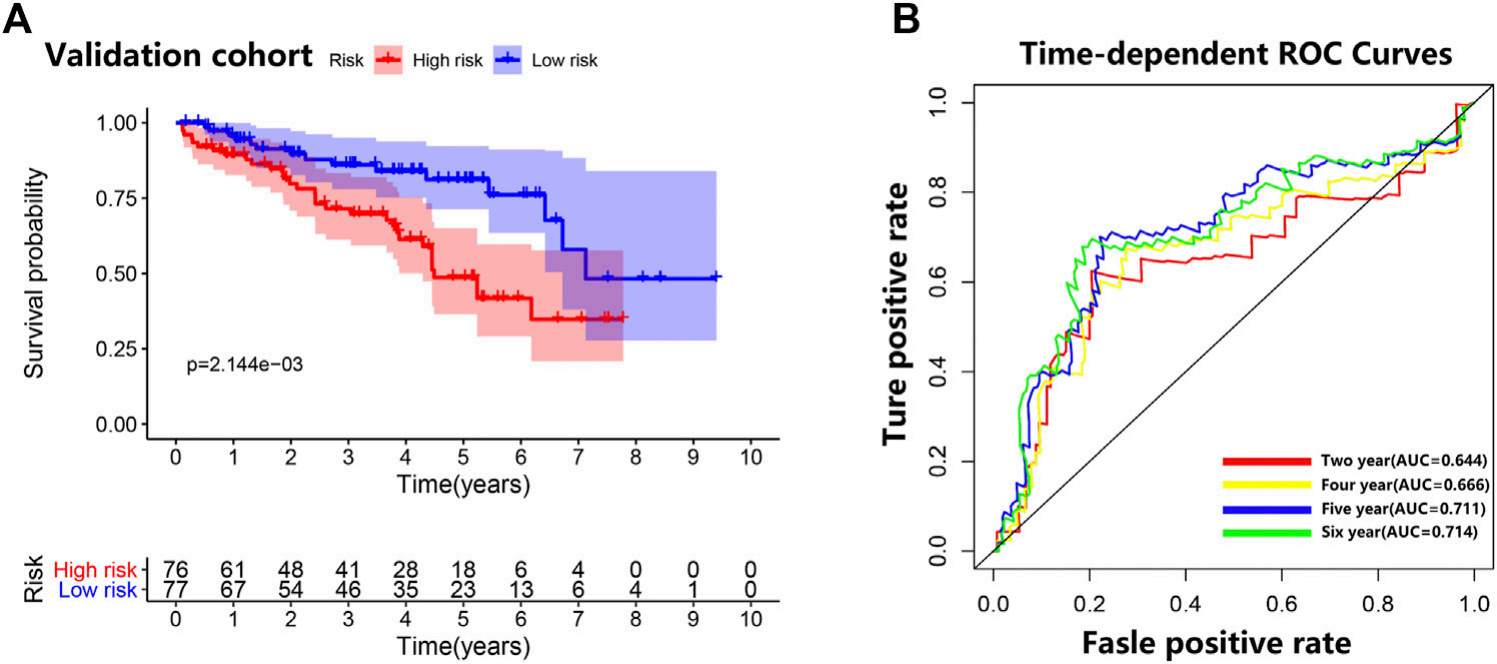
Validation cohort. **(A)** Survival curve for low-risk and high-risk subgroups in the validation cohort. **(B)** Time-dependent receiver operating characteristic curve comparison of the validation cohort. Areas under the curve (AUCs) at 2, 4, 5, and 6 years were calculated.

Consistent with the results of the training cohort, the low-risk group in the validation cohort had a better prognosis than the high-risk group did (Figure 6A). The area under the curve (AUC) (95% CI) values of the model based on the five selected genes at 2, 4, 5, and 6 years were 0.644, 0.666, 0.711, and 0.714, respectively (Figure 6B), indicating that the model achieved good sensitivity and specificity for survival prediction.

### Internal Validation With the Clinical Survival Prediction Model

**FIGURE 7:**
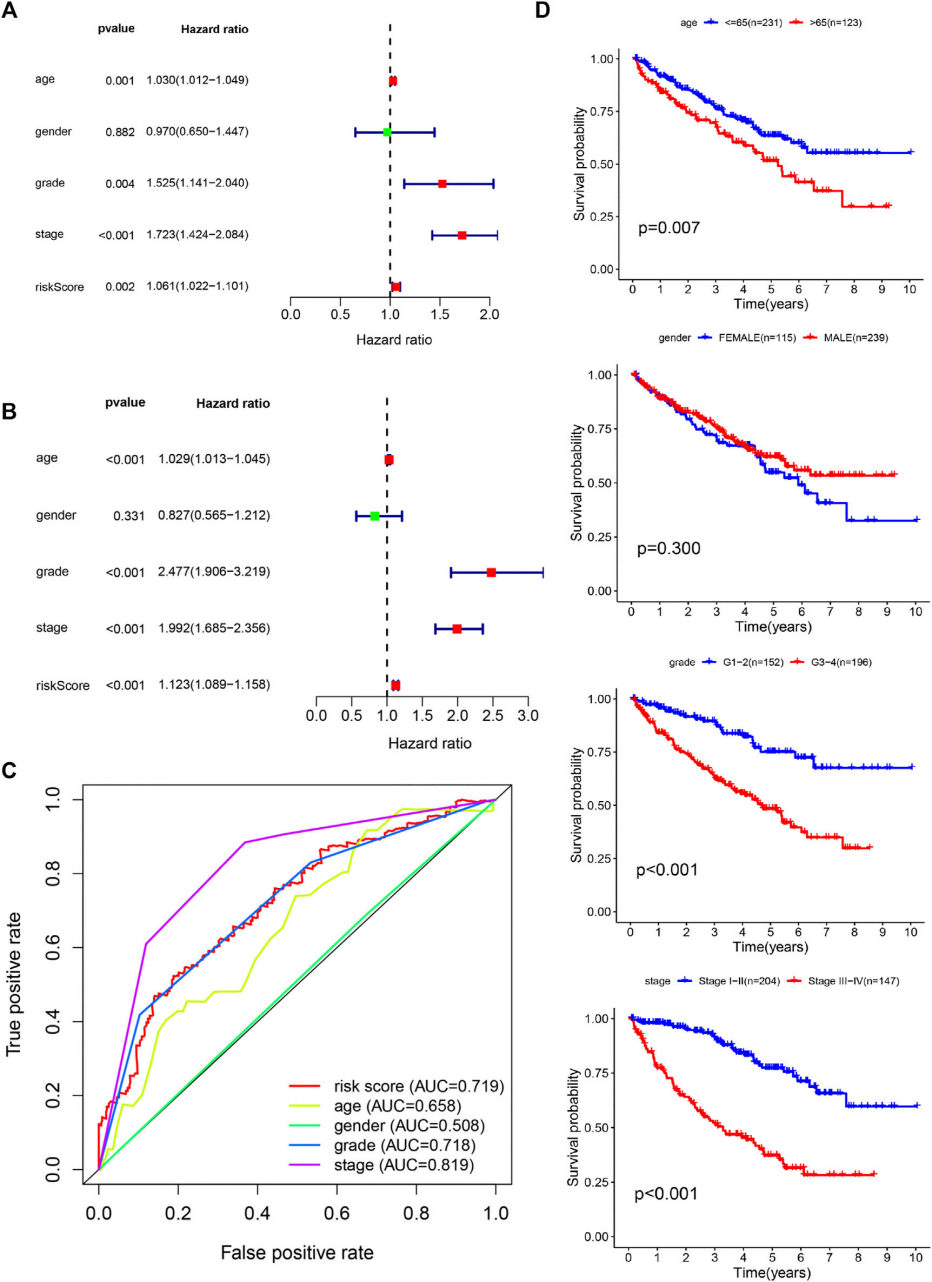
Confirmation of prognostic value and independent predictive power in patients with clear cell renal cell carcinoma (ccRCC). **(A)** Multivariate Cox analysis. **(B)** Univariate Cox analysis. **(C)** Comparison of receiver operating characteristic curves with other common clinical characteristics shows the superiority of the risk score. **(D)** Overall survival analysis based on clinicopathologic parameters.

The clinicopathologic characteristics of patients are listed in Table 2. We performed survival analyses on the subtype clinicopathologic parameters for patients in both groups. The overall survival of the high-risk group was significantly lower than that of the low-risk group (*p* value < 0.05) (Figure 4C). Kaplan-Meier plots were generated in digital form, highlighting the prognostic value of various clinical variables, and demonstrating that the data proved to be reasonable and valid. The prognostic performance of the five genes was closely related to prognosis and an AUC value of 0.719 in the model indicated a good prognostic prediction effect (Figure 7C).

**TABLE 2:** The clinical characteristic information of all patients with clear cell renal cell carcinoma (ccRCC) in The Cancer Genome Atlas database.

Variables	TCGA	Training group	Validation group
Number of Patients	507	354	153
Age			
<65	323	222	101
≥65	184	132	52
Gender			
Female	174	115	59
Male	333	239	94
Survival Status			
Alive	345	239	106
Dead	162	115	47
Grade			
G1	12	8	4
G2	215	144	71
G3	199	148	51
G4	73	48	25
GX	5	5	0
Unknow	3	1	2
Stage			
I	253	169	84
II	53	35	18
III	116	88	28
IV	82	59	23
Unknow	3	3	0
T classification			
T1	259	173	86
T2	65	44	21
T3	172	128	44
T4	11	9	2
M classification			
M0	401	278	123
M1	78	56	22
MX	26	18	8
Unknow	2	2	0
N classification			
N0	225	152	73
N1	16	13	7
NX	266	189	77

**FIGURE 8:**
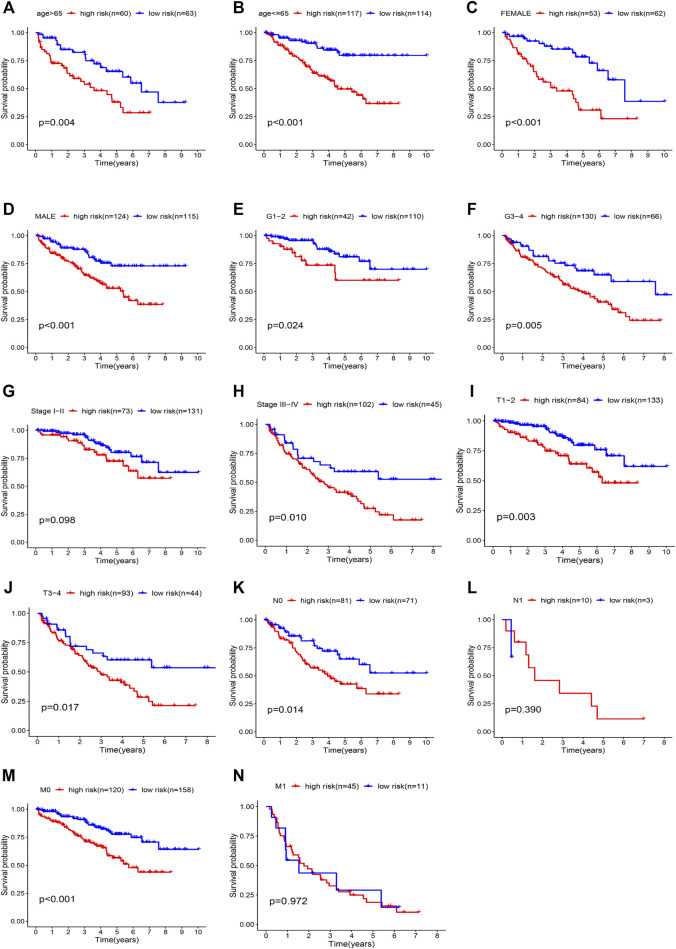
Kaplan-Meier curves showing the prognostic prediction performance in subgroups of **(A, B)** age, **(C, D)** gender, **(E, F)** grade, **(G, H)** tumor stage, **(I, J)** T stage, **(K, L)** N stage, and **(M, N)** M stage.

The model of hypoxia- and immune-related genes was significantly superior to traditional clinical factors, such as age, gender, and tumor grade (AUC values of 0.658, 0.665, 0.508, and 0.718), in the ability to predict prognosis (Figure 7C). Univariate Cox regression analysis confirmed these observations (Figure 7A). Analysis showed that age, tumor grade, tumor stage, and corresponding risk score were clinicopathologic characteristics associated with overall survival. In addition, multivariate Cox regression analysis (Figure 7B) confirmed that age, tumor grade, tumor stage, and risk score were four independent prognostic factors associated with poor overall survival (Figures 8A,B). These results confirmed that the findings of this study are based on an actual signal in the data with HRGs and IRGs and are not driven by clinical bias.

Stratified analysis was then performed in different subgroups to re-confirm the prognostic characteristics. Prognostic markers differed significantly in most subgroups (Figures 8C-K,M, *p* value < 0.05), but the results were less satisfactory in the N1 and M1 subgroups (Figures 8L,N). The results showed that the five characteristic models of hypoxia- and immune-related genes had a good predictive effect for the prognosis of ccRCC.

### Compositions of Tumor-Infiltrating Immune Cells in Patients With Clear Cell Renal Cell Carcinoma

**FIGURE 9:**
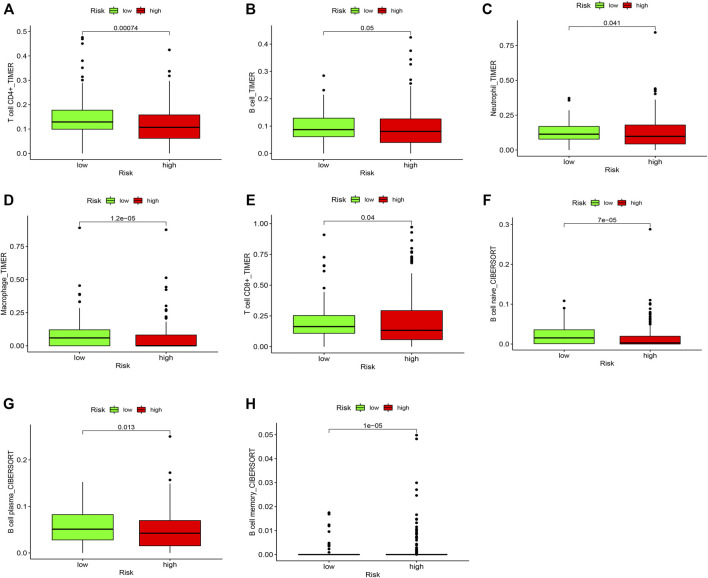
Comparison of the proportions of tumor-infiltrating immune cells between low and high risk groups. The green boxplots represent the low-risk group, and the red boxplots represent the high-risk group. **(A)** CD4^+^ T cell, **(B)** B cell, **(C)** Neutrophil, **(D)** Macrophage, **(E)** CD8^+^ T cell, **(F)** Naive B cell, **(G)** Plasma B cell, and **(H)** Memory B cell.

We compared the proportions of tumor-infiltrating immune cells between ccRCC and normal samples (Supplementary Figure S1). The results showed a significant difference in ccRCC and normal samples (Supplementary Figure S2). Furthermore, we investigated the level of infiltration of seven immune cell types to explore the relationship between risk score and infiltrating immune cell subtypes. The results showed a positive correlation between the high-risk group and the infiltrating immune cells at the tumor site, specifically memory B cells (Figure 9H). However, the high-risk group negatively correlated with CD4^+^ T cells, B cells, neutrophils, macrophages, CD8^+^ T cells, naive B cells, and plasma B cells, and the low-risk score was likely to be accompanied by many immune cell infiltrations (Figures 9A–G). These results suggest that prognostic characteristics may affect the prognosis of patients with ccRCC by regulating the tumor immune microenvironment.

### Establishment of a Nomogram Based on Risk Score and Clinicopathological Factors

**FIGURE 10:**
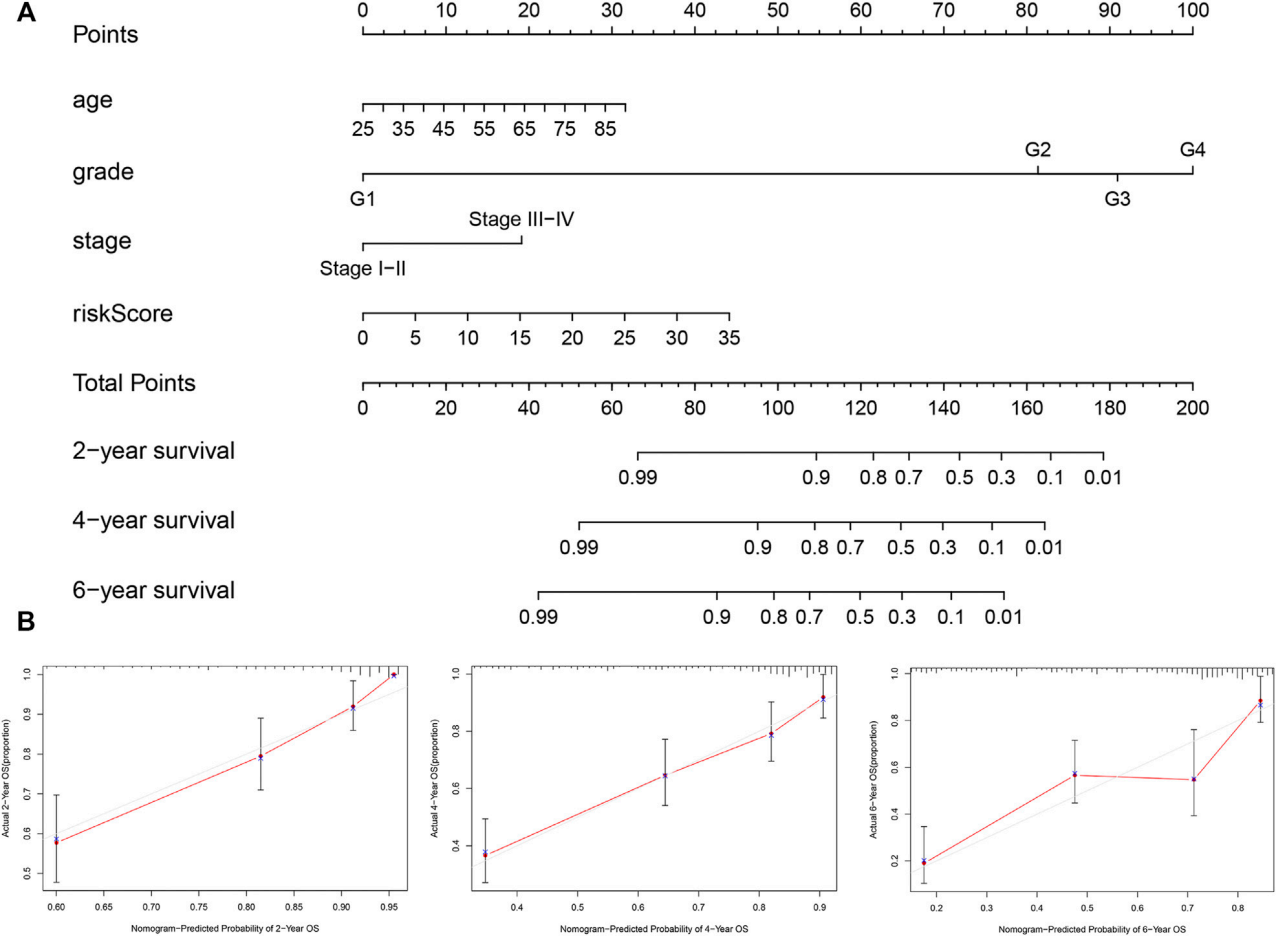
Nomogram and calibration plots for prediction of patients’ survival in the entire TCGA set. **(A)** Nomogram combining the five hypoxia- and immune-related genes risk signature with clinical factors for prediction of 2-year, 4-year, and 6-year survival rates. **(B)** Calibration plots showing high predictive accuracy of the nomogram.

Based on the outcomes of multivariate analysis of entire TCGA cohort, we constructed a prognostic nomogram to develop a more convenient and qualitative predictive tool that can predict the survival risk of individual patients (Figure 10A). In addition, the 2-, 4- and 6-year calibration curves were plotted, respectively (Figure 10B), which showed a good consistency between the predicted and actual survival rates of patients with ccRCC in the entire TCGA cohort.

### Validation of Candidate Genes by qRT-PCR

**FIGURE 11:**
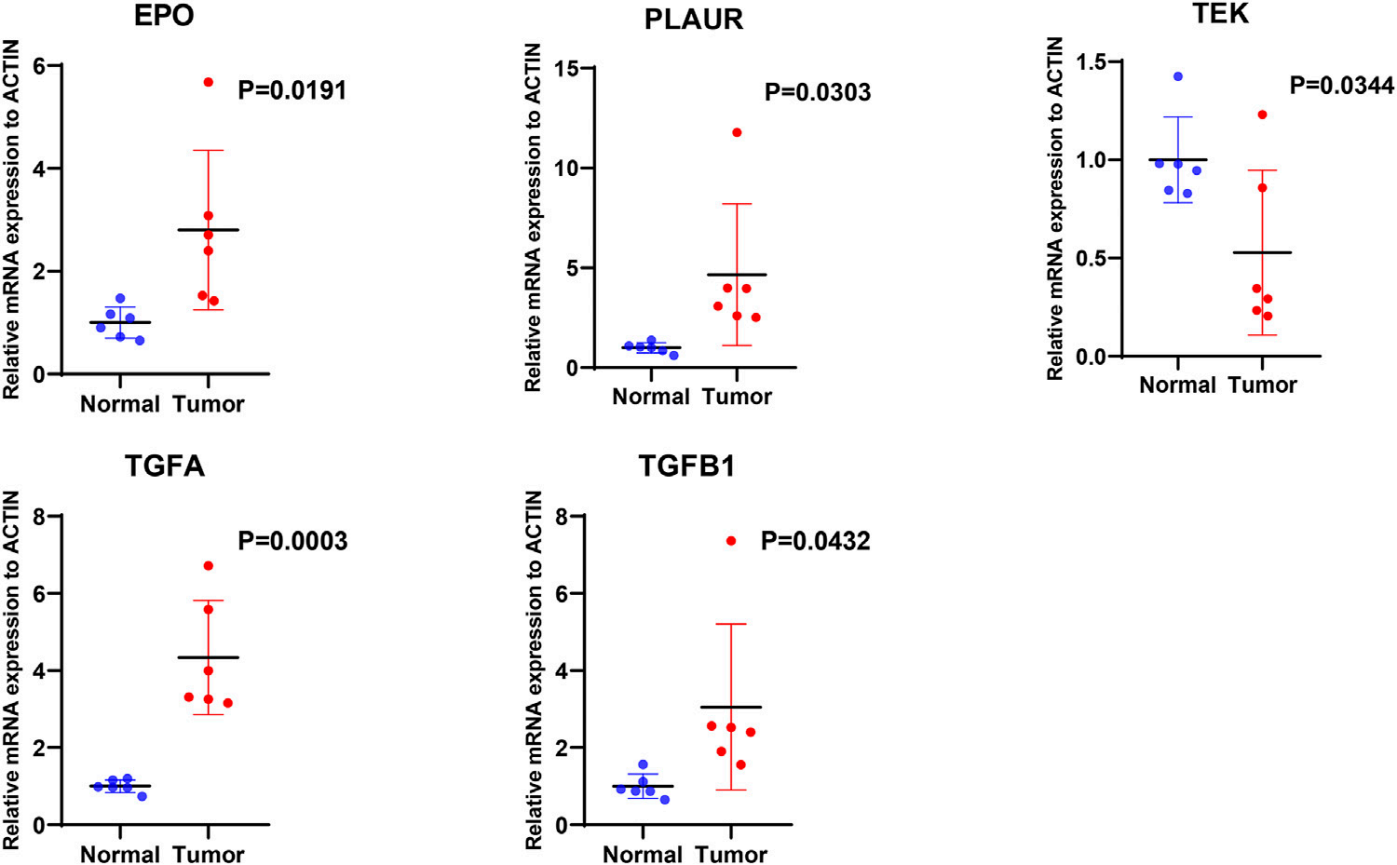
The mRNA expression levels of five hypoxia- and immune-related genes were evaluated using qRT-PCR in ccRCC samples and normal samples.

We further examined the differential expression of *EPO, TEK, TGFA, TGFB1* and *PLAUR* genes between ccRCC tissue and normal renal tissue samples. The qRT-PCR results showed that compared with the normal renal tissues, the expression level of *EPO, PLAUR, TGFA* and *TGFB1* were higher in the ccRCC tissue, while the expression level of *TEK* were lower, trends in the expression levels of these genes were consistent with our findings (Figure 11).

## Discussion

Thus far, most research on RCC has focused on the ccRCC subtype. More than 90% of chronic RCC diagnoses are characterized by loss of heterozygosity on the short arm of chromosome 3 (Gnarra et al., 1994). Approximately 50% of the cases have gene mutations (Schraml et al., 2002), whereas 5–10% of cases have promoter hypermethylation, leading to accumulation of HIF and overexpression of many genes, including those that promote angiogenesis and metabolic reprogramming (Godlewski et al., 2017).

In this study, we conducted a bioinformatics analysis based on a public database and found 30 differentially expressed hypoxia- and immune-related genes in patients with ccRCC. After conducting multiple Cox regression analyses, we identified five independent prognostic genes: *EPO*, *TEK*, *TGFA*, *TGFB1, PLAUR*. Based on these results, we developed a new prognostic model for predicting the overall survival of ccRCC patients. In addition, we validated the model and obtained consistent results, suggesting that this hypoxia- and immune-related gene signature can be used as a prognostic marker for ccRCC.

Among the five genes we obtained, the *EPO* gene is involved in the regulation of human classical physiologic response to hypoxia, and the study of its regulation led to the discovery of a human oxygen sensing mechanism (Schodel and Ratcliffe, 2019). It has been reported that *EPO* is highly expressed in RCC and is directly controlled by hypoxia via HIF-1. HIF-1 DNA is a *trans* acting factor and binds to the *cis*-hypoxia response element of the *EPO* gene promoter (Semenza 1998; Papworth et al., 2009; Masson and Ratcliffe, 2014). The erythropoietin it encodes is an erythropoietic growth factor, which can not only stimulate angiogenesis (Hardee et al., 2006) but also stimulate the proliferation of tumor cells (Hardee et al., 2006). In addition, studies have shown that human renal cancer cells express the EPO receptor, which, when activated, can stimulate the proliferation of cultured renal cancer cells *in vitro* (Hardee et al., 2006).

*TEK* was originally thought to be a specific receptor for endothelial cells, which plays an important role in the regulation of angiogenesis and remodeling and influences the formation of the tumor microenvironment (Chen et al., 2021). Alterations in *TEK* expression have been observed in many cancers, such as oral squamous cell carcinoma; leukemia; and breast, gastric, and thyroid cancers (Mitsutake et al., 2002; He et al., 2015; Chen et al., 2016; Cortes-Santiago et al., 2016; Kitajima et al., 2016). Recent studies have reported that high *TEK* expression be related to poor prognosis in patients with ccRCC, and these reports conform with the results of our study (Ha et al., 2019).

Transforming growth factor-α (TGFA), as a member of the epidermal growth factor receptor family, is believed to be an important mediator in tumorigenesis and malignant progression (Holbro et al., 2003; Hynes and Lane, 2005; Asami and Atagi, 2014). Transforming growth factor-α/epidermal growth factor receptor signaling promotes the occurrence and progression of cancer cells and generates a tumor microenvironment advantageous to metastasis (Sasaki et al., 2013). The regulation of autocrine signaling by transforming growth factor-α ligand through the epidermal growth factor receptor is also involved in the development and progression of epithelial tumors (Sporn and Todaro, 1980; Sporn and Roberts, 1985).

The transforming growth factor-β superfamily is a group of multifunctional cytokines involved in cell proliferation and differentiation, angiogenesis, immunosuppression, cell motility, apoptosis, wound healing, and embryonic development (Katz et al., 2013). Of the three TGFB isoforms that exist in humans, TGFB1 is the most abundant (Zu et al., 2012). It is encoded by the *TGFB1* gene on chromosome 19q13.2 and is associated with susceptibility to cancer. TGFB was confirmed as a promoter of the invasion and metastasis of tumor cells by regulating the immune system and the tumor microenvironment (Mishra et al., 2005; Massague 2008). TGFB1 has enhanced the proliferation and metastatic potential of renal carcinoma by upregulating lymphoid enhancer-binding factor 1/integrin αMβ2 (Liu and Shang, 2020).

The protein encoded by the *PLAUR* gene is the receptor of PLAU (plasminogen activator, urokinase), which plays a momentous role in the migration and proliferation of tumor cells through remodeling of the extracellular matrix and the tumor microenvironment (Grismayer et al., 2012; Hakelius et al., 2013; Narayanaswamy et al., 2016). In addition, *PLAUR*-mediated PLAU signal transduction activation effects are independent of proteolysis through ITGB1 and vascular endothelial growth factor receptor 2 (Larusch et al., 2013) and modulate single-chain PLAU–mediated angiogenesis.

In conclusion, we used public databases to develop a risk scoring model based on five hypoxia- and immune-related genes as potential features reflecting the prognosis of ccRCC. Compared to several researchers already established and validated signatures (Ghatalia et al., 2019; Hua et al., 2020), our model contains not only immune-related genes, but also hypoxia-related genes. Current evidence suggests that show that hypoxia and hypoxia-related pathways play critical roles in the occurrence and progress of renal cancer (Gossage et al., 2015; Choueiri and Kaelin, 2020). Most ccRCC are associated with loss of von Hippel-Lindau tumor suppressor (pVHL) function and deregulation of hypoxia pathways (Schodel et al., 2016). Targeting the HIF2-Vascular endothelial growth factor (VEGF) axis, multiple VEGF inhibitors are approved for the treatment of ccRCC, and a HIF2α inhibitor has advanced to phase 3 development for this disease (Choueiri and Kaelin, 2020). Therefore, our results are more closely related to the mechanisms of ccRCC development and clinical treatment applications, and can provide a better perspective for ccRCC research and personalized prediction. However, more clinical trials are needed to verify our observations, and additional experimental studies must verify the potential mechanism behind the predictive value of this hypoxia- and immune-related gene signature in ccRCC.

## Data Availability

The datasets presented in this study can be found in online repositories. The names of the repository/repositories and accession number(s) can be found in the article/Supplementary Material.
