# Supplementary material for: Construction and Verification of a Combined Hypoxia and Immune Index for Clear Cell Renal Cell Carcinoma
Source: Front Genet. 2022 Feb 9;13:711142. doi: 10.3389/fgene.2022.711142 (PMC8863964; doi:10.3389/fgene.2022.711142)
Supplement: Supplementary file 5 [file Image2.pdf]

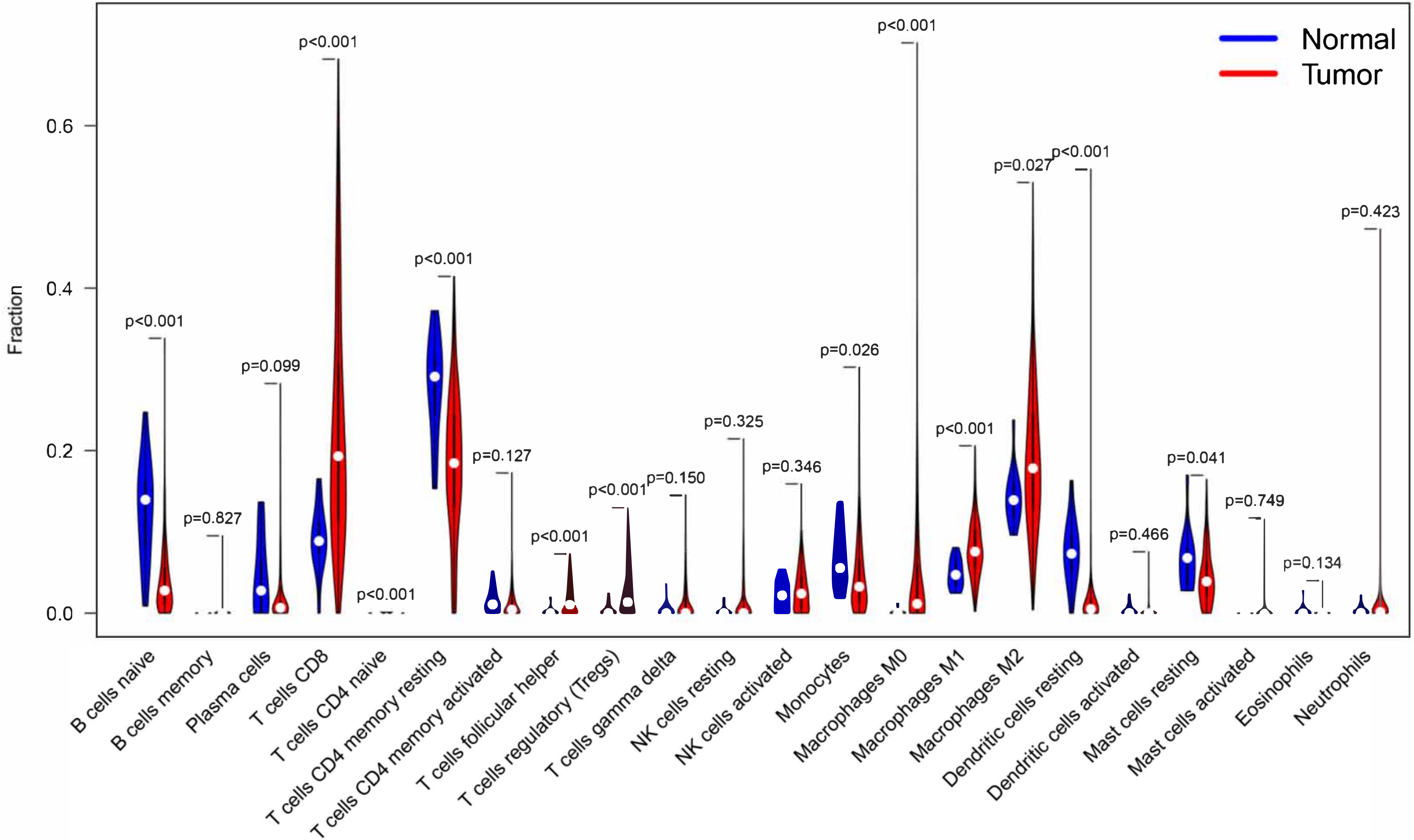

Supplementary Figure2: Violin plot comparing the proportions of TIICs between ccRCC and Normal samples. Horizontal and vertical axes respectively represent TIICs and relative percentages. Blue and red colors represent low and high immune score ccRCC samples, respectively. Data were assessed by the Wilcoxon rank-sum test. (*P<0.05, **P<0.01, ***P<0.001. NS, no significance; NK, natural killer.)
