# Supplementary material for: Construction and Verification of a Combined Hypoxia and Immune Index for Clear Cell Renal Cell Carcinoma
Source: Front Genet. 2022 Feb 9;13:711142. doi: 10.3389/fgene.2022.711142 (PMC8863964; doi:10.3389/fgene.2022.711142)
Supplement: Supplementary file 6 [file Table2.DOCX]

Supplementary Table 2. Primer sequences used in this study.

| **Genes** | **Primers (5’-3’)** |
| --- | --- |
| EPO-F | GGAGGCCGAGAATATCACGAC |
| EPO-R | CCCTGCCAGACTTCTACGG |
| TEK-F | TTAGCCAGCTTAGTTCTCTGTGG |
| TEK-R | AGCATCAGATACAAGAGGTAGGG |
| TGFA -F | AGGTCCGAAAACACTGTGAGT |
| TGFA -R | AGCAAGCGGTTCTTCCCTTC |
| TGFB1-F | GGCCAGATCCTGTCCAAGC |
| TGFB1-R | GTGGGTTTCCACCATTAGCAC |
| PLAUR -F | TGTAAGACCAACGGGGATTGC |
| PLAUR -R | AGCCAGTCCGATAGCTCAGG |
| β-actin -F | CATGTACGTTGCTATCCAGGC |
| β-actin -R | CTCCTTAATGTCACGCACGAT |
