# Supplementary material for: Construction and Verification of a Combined Hypoxia and Immune Index for Clear Cell Renal Cell Carcinoma
Source: Front Genet. 2022 Feb 9;13:711142. doi: 10.3389/fgene.2022.711142 (PMC8863964; doi:10.3389/fgene.2022.711142)
Supplement: Supplementary file 7 [file Image1.pdf]

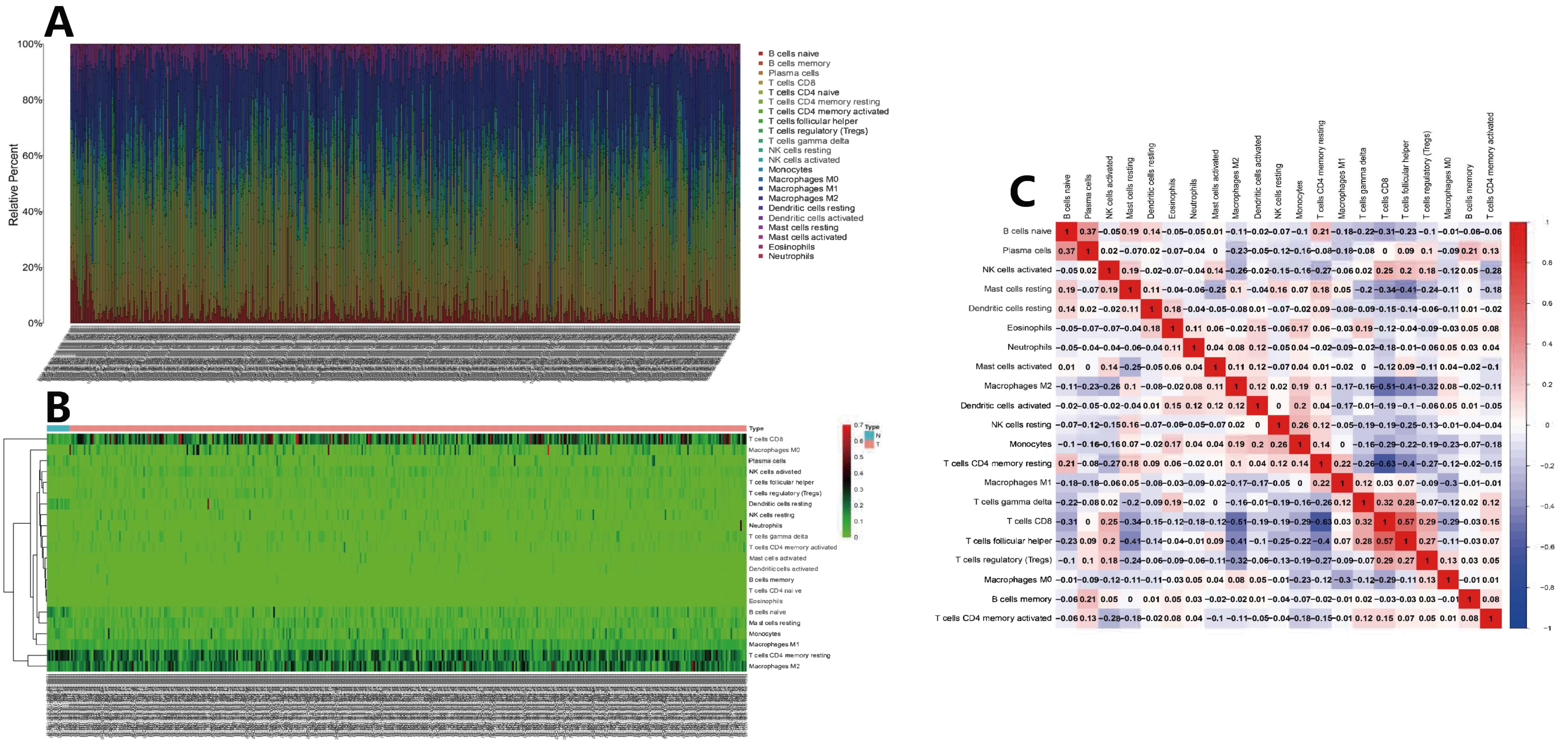

Supplementary Figure1: The landscape of immune infiltration in ccRCC. (A)The difference of immune infiltration in each sample of ccRCC and normal tissues. (B)Heat map of the 22 immune cell proportions. (C)Correlation matrix of all 21 immune cell densities in the TCGA cohort.(Horizontal and vertical axes both represent TIICs. TIICs with higher, lower, and same correlation levels are shown in red, blue, and white, respectively. TIIC, tumor-infiltrating immune cell.)
